# Supplementary material for: The genome sequence of the Hebrew Character, Orthosia gothica (Linnaeus, 1758)
Source: Wellcome Open Res. 2024 Feb 19;9:90. [Version 1] doi: 10.12688/wellcomeopenres.20904.1 (PMC11487229; doi:10.12688/wellcomeopenres.20904.1)
Supplement: Peer review history

# Reviewer report: Reviewer response for version 1

**DOI:** 10.21956/wellcomeopenres.23130.r99894

**Published:** 2024-10-17

**Authors:** Alessandro Grapputo

**Affiliations:** 1 Dipartimento di Biologia, Universita degli Studi di Padova, Padua, Veneto, Italy

The paper by Boyes and colleagues present the reference genome of the Hebrew Character moth *Orthosia gothica* a spring-flying moth found across Eurasiaa. The data are well presented and accessible. The assembled genome is of high quality representing the reference genome for the specie. The paper is well written. The methods of DNA/RNA extraction, sequence acquisition, genome assembly pipeline and annotation with the Ensembl rapid annotation pipeline follow the classic protocol of most Data Note papers published by Wellcome Open Research. In summary the note is sound and  good to be indexed.

Are sufficient details of methods and materials provided to allow replication by others?

Yes

Is the rationale for creating the dataset(s) clearly described?

Yes

Are the datasets clearly presented in a useable and accessible format?

Yes

Are the protocols appropriate and is the work technically sound?

Yes

Reviewer Expertise:

Molecular ecology

I confirm that I have read this submission and believe that I have an appropriate level of expertise to confirm that it is of an acceptable scientific standard.

# Reviewer report: Reviewer response for version 1

**DOI:** 10.21956/wellcomeopenres.23130.r91876

**Published:** 2024-09-10

**Authors:** Ricardo Rodriguez-de-la-vega

**Affiliations:** 1 Écologie Systématique et Évolution, CNRS, Université Paris-Saclay, Paris, France

Comments on Boyes et al., "The genome sequence of the Hebrew character..."

 In this data note, the Darwin Tree of Life's inordinate fondness for moths continues with the release of what is approximately the 314th moth genome*.

 As is typical for the consortium’s outputs, the combination of HiFi and Hi-C sequencing (both from the same specimen in this case) and manual curation has produced a chromosome-level assembly, which can be reused for further analysis. While the genome excels in all metrics presented in the data note, I would encourage readers to explore the extensive online materials for a more nuanced understanding of the genome's quality and specific features. To point out just two: heterozygosity seems unusually high, and the Hi-C fragment separation distribution is closer to the "bad example" when compared to other genome assemblies within the Noctuidae family.

 A telling detail, only briefly mentioned in the hyper-standardised wording common in DToL data notes, is that the sequenced individual was a female, alas no W chromosome was identified, even though combined HiFi and Hi-C placed over 97.6% of the assembled contigs onto chromosomes.

 Another not discussed point is about the large contig (11 Mb) with twice as much coverage as the chromosome anchored contigs in the GC-coverage, which might be a cobiont. The DToL consortium might need to consider updating their Data Note standards to start discussing these no-hit contigs.

 Mind that in the text, the Z chromosome is said to have been identified based on coverage, but Table 1 suggests identification by homologous pairs.

 I would like to thank the people in charge of peer reviewers assignments and DToL's commitment to presenting common names for giving me the opportunity to discover the world of butterfly/moth vernacular naming in the UK.

 *DToL gateway queried on August 30th, 2024.

Are sufficient details of methods and materials provided to allow replication by others?

Yes

Is the rationale for creating the dataset(s) clearly described?

Yes

Are the datasets clearly presented in a useable and accessible format?

Yes

Are the protocols appropriate and is the work technically sound?

Yes

Reviewer Expertise:

Evolutionary genomics, bioinformatics

I confirm that I have read this submission and believe that I have an appropriate level of expertise to confirm that it is of an acceptable scientific standard.
